# Resting-State Electroencephalography for Prognosis in Disorders of Consciousness Following Traumatic Brain Injury

**DOI:** 10.3389/fneur.2020.586945

**Published:** 2020-12-04

**Authors:** Ruth Pauli, Alice O'Donnell, Damian Cruse

**Affiliations:** Centre for Human Brain Health, University of Birmingham, Birmingham, United Kingdom

**Keywords:** resting-state EEG, disorders of consciousness, prognosis, traumatic brain injury, coma

## Abstract

Although the majority of patients recover consciousness after a traumatic brain injury (TBI), a minority develop a prolonged disorder of consciousness, which may never fully resolve. For these patients, accurate prognostication is essential to treatment decisions and long-term care planning. In this review, we evaluate the use of resting-state electroencephalography (EEG) as a prognostic measure in disorders of consciousness following TBI. We highlight that routine clinical EEG recordings have prognostic utility in the short to medium term. In particular, measures of alpha power and variability are indicative of relatively better functional outcomes within the first year post-TBI. This is hypothesized to reflect intact thalamocortical loops, and thus the potential for recovery of consciousness even in the apparent absence of current consciousness. However, there is a lack of research into the use of resting-state EEG for predicting longer-term recovery following TBI. We conclude that, given the potential for patients to demonstrate improvements in consciousness and functional capacity even years after TBI, a research focus on EEG-augmented prognostication in very long-term disorders of consciousness is now required.

## Introduction

Traumatic brain injury (TBI) is a major cause of death and disability worldwide (1–3). The majority of people who suffer severe TBI will develop long term, and often profound, disability (3, 4), although the prognosis is nonetheless better than for anoxic brain injuries (5, 6). Outcomes of severe TBI are highly variable, and accurate prognostication remains a challenge (7). Prognosis is a particularly important consideration for patients who develop disorders of consciousness (DOCs) following TBI. These include acute coma, as well as long-term states of impaired consciousness, which can be permanent (8). While recovery is unlikely beyond the first year post-injury, multiple exceptions have been reported (9). This uncertainty about the potential for meaningful recovery can affect decisions regarding care and rehabilitation (8). Consequently, recent years have seen a drive toward the use of neuroimaging for more accurate prognostication (10, 11). Indeed, given the inherent uncertainty in measuring consciousness itself, neuroimaging markers of functional prognosis have clinical value even when they do not allow strong conclusions about conscious state directly.

Electroencephalography (EEG) is perhaps the most practical and cost-effective neuroimaging tool since it can be administered at the bedside. Although not currently part of the UK guidelines for assessment post-TBI (12), EEG has often been used to assess depth of coma [e.g., (13)]. The prognostic utility of active EEG (e.g., event related potentials) has already been demonstrated [e.g., (14, 15)], but active methods often involve procedures that are not part of routine EEG monitoring or that require additional specialist equipment (e.g., administering auditory or tactile stimuli under controlled conditions). Resting-state EEG, therefore, provides an efficient and inexpensive snapshot of cerebral health.

The most common resting-state measures are spectral measures, which quantify the amplitude of the four canonical EEG frequency bands: beta (>13 Hz), alpha (8–13 Hz), theta (4–7 Hz), and delta (0.5–3 Hz). Associations between spectral measures and level of consciousness are well-established, with higher frequencies (especially alpha) positively associated with consciousness level and lower frequencies (especially delta) negatively associated with consciousness level [e.g., (16, 17)]. Less commonly used are connectivity measures (i.e., functional connectivity between frequency bands in different brain regions), and mixed measures derived in part from spectral measures [e.g., (13)]. Although not purely resting-state measures, we include these mixed measures because they were designed specifically for prognostic purposes, and rely only on routine bedside EEG recordings. The majority of the literature we review here focuses on relatively short-term prognosis in acute coma (typically 3–12 months post-injury outcome). A minority of studies have investigated longer term (>12 months) prognosis, and a smaller number still have investigated prognosis in chronic DOCs, when EEG measurements are taken weeks, months, or years after the initial trauma.

The aim of this review is to evaluate the potential for resting-state EEG to be used as a prognostic tool in DOCs following TBI, and to identify priorities for future research, such as specific EEG features and the clinical populations most likely to benefit from EEG-augmented prognosis, before resting-state EEG can be considered for use in clinical practice. We focus on measures that can be collected during routine clinical EEG. We begin this review by briefly introducing the various DOC diagnoses and the common measures of consciousness and functional recovery. We then review the prognosis for recovery from coma, first in the short and then in the longer term, before reviewing the few studies that have investigated prognosis in chronic DOCs. We conclude by highlighting the disparities in focus on these different stages of recovery, and stressing the need to address these disparities as an important future direction for research into chronic DOCs.

## Methods

We first used the online database Scopus and Google Scholar, using the search terms “EEG” or “electroencephalogram,” “coma” or “disorders of consciousness,” and “prognos^*^,” “recover^*^,” or “predict^*^.” We did not specify the year of publication. This search generated 1,616 papers, of which 1,537 were excluded based on the title and abstract because they were not relevant (e.g., did not relate to prognosis or DOCs, did not use EEG, focused on anoxic etiology only). A further 65 articles were rejected after careful reading because the full text was not available in English, or because the authors did not present separate results for TBI and non-TBI groups, did not use resting-state EEG, or focused on sleep. We conducted an additional search of PubMed with the search terms “vegetative state” or “minimally conscious state” in place of “disorders of consciousness” (as above), which did not generate any additional papers with a specific prognostic relevance. The 14 papers included in the review are summarized in Table 1. We conducted this project under the principles of the Declaration of Helsinki.

**Table 1:** Overview of research papers included in this review.

**Author, date of publication**	**N (TBI only)**	**Time of initial EEG (post-TBI)**	**Initial diagnosis**	**Time of outcome assessment (post-TBI)**	**Outcome measure**	**EEG measures**	**Main findings**
Moulton et al. (18)	103	0–5 days	Coma	Within 5 days	GCS (good, moderate, poor)	Spectral measures (power)	EEG did not predict outcome
Steudel and Krüger, (19)	50	0–7 days	Coma	6 months	Survival (alive at 6 months/deceased between 1 week and 6 months post-TBI)	Spectral measures (power) over parieto-occipital cortex	Parieto-occipital percentage alpha and theta power positively predicted survival
Vespa et al. (20)	89	0–10 days	Coma	30 days	GOS [good (4–5)/poor (1–2)]	Percentage alpha variability	High or increasing percentage alpha variability associated with better outcome
Hebb et al. (21)	53	0–7 days	Coma	6 months	GOS scores	Percentage alpha variability	Higher percentage alpha variability predicted better outcome
Beridze et al. (22)	53	0–5 days	Coma	12 months	Deceased, UWS, or recovered	Spectral measures (various)	Delta coma predicted UWS and death
Schnakers et al. (23)	13	0–1 days	Coma	6 months	GOS-E scores	Spectral measures (various)	Alpha power positively predicted better outcome
Tolonen et al. (24)	28	2–5 days	Coma	6–12 months	GOS-E [favorable (4–5)/unfavorable (1–3)]	Spectral measures (various)	Median alpha power, relative fast theta power variability, and relative alpha power variability best predicted favorable outcome
Kane et al. (25)	60	1–10 days	Coma	12 months	DRS scores	Spectral measures across various brain regions	Power in all canonical bands predicted good outcome
Rae-Grant et al. (26)	68	2–5 days	Coma	6 months	GOS scores	Scores based on various components, including spectral measures	EEG scores predicted outcome better than other neurophysiological measures, but no better than GCS scores
Bagnato et al. (27)	22	14–0 days	Coma	3 months post-admission (~3.5–6 months post-TBI)	LCFS scores (change from baseline)	Synek grades	Synek grades predicted less recovery between admission and follow-up
Fingelkurts et al. (28)	3	3 months	UWS	6 years	Diagnosis (UWS/MCS/E-MCS)	Index of Operational Synchrony	Alpha and beta synchrony predicted transition to MCS or E-MCS
Schorr et al. (29)	14	30 days−12 years	UWS	12 months post-baseline (~1–12 years post-TBI)	CRS-R (diagnosis)	Coherence	Patients who remained in UWS had higher fronto-parietal and fronto-occipital coherence in gamma band, compared to recovered patients
Bareham et al. (30)	4	Regularly for up to 2 years, starting 19 months−9 years post-TBI	UWS (2) or MCS- (2)	2 years post-baseline (~3–11 years post-TBI)	CRS-R (diagnosis)	Network metrics—different for each patient	In patients whose diagnosis changed, recovery correlated positively with normalized alpha band participation coefficient (patient 1), and negatively with delta power (patient 3)

## Disorders of Consciousness and Measures of Recovery

Consciousness is medically defined along two dimensions: arousal (or wakefulness) and awareness (31). Coma is a lack of arousal and awareness that lasts for at least 1 h, and rarely more than a few days or weeks. A patient in coma does not exhibit a sleep-wake cycle and cannot be aroused, as evidenced by a lack of eye-opening to stimulation. Unresponsive wakefulness syndrome (UWS; also referred to as vegetative state) is diagnosed when a patient emerges from coma to the extent that they can be aroused, exhibit a sleep-wake cycle, and might spontaneously open their eyes, but do not exhibit signs of awareness, such as voluntary motor movements. Any movements in UWS are reflexive only. Finally, in a minimally conscious state (MCS), the patient exhibits awareness as demonstrated by purposeful behavior, such as visual tracking of salient stimuli, responding to commands, and laughing or crying in response to humorous or sad situations (31). MCS is sometimes further divided into MCS+ and MCS–, with the former characterized by higher-level behaviors such as command following, and the latter characterized by lower-level behaviors such as visual pursuit (32). Patients are considered to be emerging from an MCS (E-MCS) when they demonstrate either consistent functional communication, or functional use of at least two objects (31).

The Glasgow Coma Scale (GCS) is the standard clinical tool for assessing neurological functioning immediately after brain injury (33–35). The Glasgow Outcome Scale (GOS) and Glasgow Outcome Scale—Extended (GOS-E) are common measures of functional recovery, typically administered within the first 3–12 months after injury (4, 36, 37). Other measures cited in this literature review are the Disability Rating Scale [DRS; (38)], the Levels of Cognitive Functioning Scale [LCFS; (39)], and the Coma Recovery Scale Revised [CRS-R; (40)]. These measures are summarized in Table 2.

**Table 2:** Measures of consciousness and recovery.

**Measure name**	**Measure type**	**Score range**	**Lowest score indicates**	**Highest score indicates**
Glasgow Coma Scale (GCS)	Consciousness	3–15	Severe (brain death/coma)	Mild (normal/near-normal consciousness)
Level of Cognitive Functioning Scale (LCFS)	Consciousness/cognitive impairment	1–8	Unresponsive	Purposeful and appropriate behavior
Cognitive Recovery Scale Revised (CRS-R)	Consciousness/cognitive impairment	0–23	Unresponsive	Normal consciousness
Disability Rating Scale (DRS)	Consciousness/cognitive and functional impairment	0–29	Best outcome (fully conscious, normal cognitive ability, independence in daily living, employable)	Worst outcome (unconscious, severe cognitive impairment, dependent, unemployable)
Glasgow Outcome Scale (GOS)	Functional impairment	1–5	Death	Good recovery (mild neurological/psychological deficits only)
Glasgow Outcome Scale—Extended (GOS-E)	Functional impairment	1–8	Death	Upper good recovery (resumption of normal life, no deficits/non-disabling minor deficits)

## Prognosis for Recovery From Coma

Coma rarely persists for more than a few days or weeks, and if complete recovery of consciousness is not achieved within 1 year of the initial TBI, the resulting DOC is generally considered to be permanent [although this is now questioned; (9)]. Consequently, EEG recordings are usually made within hours or days of the initial injury and used to predict recovery up to, but rarely beyond, the 12-month mark. Two studies have used EEG spectral measures to predict very short-term prognosis following TBI. Moulton et al. (18) studied 103 patients with severe closed head injuries (admission GCS ≤ 8), who were monitored with EEG for ~5 days, until death, awakening from coma, or intracranial physiological stability occurred. However, EEG spectral measures did not predict good (no or moderate disability), moderate (severe disability), or poor outcomes (death or UWS), as judged by GCS scores, at the end of the monitoring period. By contrast, an earlier EEG study demonstrated that parieto-occipital percentage alpha and theta power positively predicted post-1-week survival rates in coma following TBI (19).

More commonly, outcomes have been measured 1–12 months after the initial TBI. Vespa et al. (20) analyzed percentage alpha variability (PAV) from continuous EEG recordings in 89 TBI patients (mean age 39), 0–10 days post-admission to intensive care [see also (41)]. PAV scores reflect the variability in percentage alpha power during a given time period (e.g., several hours), and are calculated from differences between the peaks and subsequent troughs in the percentage alpha power. PAV therefore reflects the rhythm of alpha power waxing and waning throughout the day, rather than a minutes-long “snapshot” of electrical activity. In this study, daily average whole brain PAV scores were calculated for each patient. GCS scores were recorded on admission and GOS scores were recorded at 30-day follow-up. Good outcomes (GOS 4-5) were associated with higher mean PAV scores in the first 3 days post-admission, while poor outcomes (GOS 1-2) were associated with lower PAV scores in the same period. Across the full period of EEG monitoring, increasing PAV scores or sustained high PAV scores were associated with better outcomes, while decreasing or sustained low PAV scores were associated with poorer outcomes. In a classification analysis based on these early recordings, poor outcomes were predicted more accurately than positive outcomes. Importantly, however, prognostication was improved by taking PAV into account alongside standard clinical indicators, such as GCS score, pupillary response to light, and patient age. Hebb et al. (21) reported similar findings in a sample of 53 patients, with post-injury PAV scores significantly predicting GOS scores at 6 months follow-up. It should be noted that both of these studies focused exclusively on alpha band measures.

Other studies have compared multiple spectral EEG measures, encompassing all four canonical frequency bands, in predicting 1–12-month outcomes. Beridze et al. (22) followed 53 patients (aged 25–55 years) from admission to an emergency department following TBI to 1-month follow-up. GCS assessments and EEG recordings were made 5 days after admission, with GCS scores ranging from 4 to 8. At follow-up, patients were classed as deceased, in UWS, or recovered, albeit with significant neurological disability remaining. There was a positive correlation between delta coma (i.e., predominantly delta band activity) at admission and occurrence of death and UWS at follow-up [see also (42)]. In a smaller sample, Schnakers et al. (23) conducted a retrospective study of 13 patients aged 21–77 years, who were admitted to hospital following severe TBI and followed for 6 months. On admission, all patients were either in coma (GCS 3-8), or partially conscious with intracranial bleeding (GCS 14), and received continuous EEG monitoring for 24 h. Summary measures of alpha power (average and variance), delta power, and delta variance generated from a principal component analysis were used to predict GOS-E scores at 6 months follow-up, at which time the mean GOS-E score was 4.5. There was a significant positive association between alpha power and GOS-E scores, and a near-significant negative association between delta variance and GOS-E, although the latter disappeared after accounting for clinically relevant covariates, such as age, sex, and sedation level. Similarly, Tolonen et al. (24) followed 28 patients from emergency admission through to follow-up, 6–12 months post-injury. EEG recordings were recorded for ~60 h, generally within 2–5 days of admission, and used to predict favorable (GOS 4-5; 10 patients) and unfavorable (GOS 1-3; 18 patients) outcomes at follow-up. All measures of absolute frequency band power were positively associated with favorable outcome, with the strongest predictor being median alpha power. All measures of frequency band variability were also positively associated with favorable outcome. The best variability predictors were relative fast theta (5–8 Hz) power variability and relative alpha power variability. By contrast, hemispheric asymmetry in EEG measures was not related to outcome. Finally, Kane et al. (25) followed 60 closed-head injury patients (aged 1–80 years) for 12 months. EEG recordings were made at 1–10 days post-injury, when all patients were in coma (GCS ≤ 8), and were used to predict DRS scores at 12-months post-injury. The mean DRS score was 6.4. Seventeen patients had died, 1 was in UWS, 11 were severely disabled, 11 were moderately disabled, and 20 had made good recoveries. There were significant negative associations between DRS scores (where higher scores signify more impairment) and power in all canonical bands, with varying topographical maxima.

Two studies used “mixed” EEG classifications or prognostic scales to predict outcome in the first post-injury year. First, Rae-Grant et al. (26) studied 68 patients (mean age 36) who were in coma 48 h after emergency admission for TBI. Patients received EEG monitoring for at least 30 min, 2–5 days post-injury. EEG recordings were scored according to the presence or absence of various EEG features, including background frequencies in the four canonical frequency bands, reactivity, variability, asymmetry, and burst suppression. GCS scores were recorded 7 days post-injury, and GOS scores were reported at 6 months follow-up. The mean GCS score was 7.68, and the mean GOS score for surviving patients at follow-up was 4.18. Outcomes for this sample were generally poor; by follow-up, six had died, one had been transferred to a different acute hospital, one had been transferred to a hospital for chronic illnesses, and four had been discharged home. EEG scores were a significant predictor of GOS scores at follow-up, outperforming all other neurophysiological measures (e.g., evoked potentials). However, EEG scores did not provide additional prognostic value compared to GCS scores alone, with which they were highly correlated. Second, Bagnato et al. (27) used Synek-graded EEG recordings to predict recovery in 22 TBI patients (mean age 30 years). Synek classifications are generated from visual inspection of the EEG and range from 0 (normal) to 5 (electrocerebral silence). Grades 1–3 reflect progressively less alpha and more delta or theta activity, while grade 4 represents other malignant patterns, such as alpha or theta coma, or epileptiform activity. EEG recordings were made on admission to a neurorehabilitation ward (14–90 days post-injury), and consciousness was assessed with the LCFS at admission and 3 months later. All patients scored 4 or below on the LCFS at admission, and all except two patients were in UWS or MCS. There was a significant negative association between Synek grades and LCFS scores at admission, as well as between Synek grades and change in LCFS scores between admission and follow-up (i.e., change in consciousness level). Notably, this was the only study to assess recovery directly, as opposed to final outcomes. Interestingly, while Synek grades correlated with both the admission LCFS scores and the change in LCFS scores in the TBI group, a non-TBI group in the same paper exhibited correlations between Synek scores and change in LCFS scores only. This suggests that the recovery metric was independently informative, and not simply a function of baseline scores.

In summary, EEG spectral measures appear to have prognostic value in determining recovery of consciousness from coma within the first year post-TBI. This is particularly true for alpha power and variability, although it should be noted that two studies included only alpha band measures from the outset (20, 21). Although these results are promising, several limitations must be noted. First, outcomes are typically short-term, even in the context of the first post-injury year. Only two studies measured outcomes at 12 months (24, 25), and the shortest “follow-up” occurred at 5 days post-injury (18), which was well within the period of initial monitoring in several studies [e.g., (24)]. Second, small sample sizes meant that outcome measures were by necessity imprecise; several studies used tri- or dichotomous outcomes only (18, 20, 22, 24). Third, only one study (20) demonstrated an additional prognostic value to EEG as compared to standard clinical indicators, while one study made the comparison but indicated no additional benefit (26). Finally, it is interesting to note that except for Bagnato et al. (27), all of these studies assessed final outcome as opposed to recovery (i.e., change from baseline to follow-up). It is therefore to some extent a matter of semantics as to whether they demonstrate diagnostic or prognostic utility of EEG, especially for very short-term outcomes.

## Prognosis for Recovery From Chronic Docs

Patients who recover from coma but remain in UWS or MCS throughout the first year post-TBI are often regarded, perhaps wrongly, as having no chance of further recovery (9). They are thus an understudied population. Nonetheless, three studies have investigated prognosis in UWS or MCS following TBI. First, Fingelkurts et al. (28) conducted a retrospective study of three patients, aged 19, 35, and 55, who were in UWS 3 months after sustaining TBIs. Consciousness was assessed with the LCFS at 3 months and 6 years post-injury, and resting state EEG recordings were conducted at 3 months post-injury. One patient remained in UWS, one recovered to MCS, and one recovered to E-MCS by the final follow-up. Alpha and beta synchrony (i.e., number and strength of functional connections across the brain) at 3 months positively predicted transition to a higher state of consciousness (MCS or E-MCS) 6 years later. Notably, however, patients who died before the follow-up were excluded from these analyses. Second, as part of a larger prospective study, Schorr et al. (29) investigated the prognostic value of resting-state EEG coherence following TBI. At baseline (which varied from 30 days to 12 years post-injury), patients underwent 5 min of resting-state EEG recording. The CRS-R was used to assess diagnosis at baseline and again 12 months later. Fourteen patients were in UWS at baseline, of whom two progressed to at least MCS at follow-up. Those who remained in UWS exhibited significantly higher baseline fronto-parietal and fronto-occipital coherence in the gamma frequency band, as compared to the improved patients. Given that only two patients improved, however, these results must be interpreted with caution. Indeed, in the full sample, which included non-TBI patients, coherence in all frequency bands and across all four cortical lobes was associated with a higher chance of recovery.

Finally, Bareham et al. (30) report results from four patients in UWS (*n* = 2) or MCS– (*n* = 2) following TBI, as part of a larger cohort with mixed etiologies (43, 44). Patients were recruited between 19 months and 9 years post-TBI, and assessed with EEG and CRS-R every 3 months for up to 2 years. Network metrics and their percentage change from baseline were calculated from the EEG recordings. For each patient, the most promising EEG metric was selected for analysis, based on the initial diagnosis and indications from previous literature. Two patients made a partial recovery during the study period, one from UWS to MCS– and one from MCS– to MCS+. The other two patients remained in UWS (*n* = 1) or MCS– (*n* = 1) throughout the study period. In the patient who progressed from UWS to MCS– (recruited 9 years post-TBI), CRS-R scores were correlated with the normalized alpha band participation coefficient (a measure of alpha network centrality). In the patient who remained in UWS (recruited 19 months post-injury), there was no change in CRS-R scores. In the patient who progressed from MCS– to MCS+ (recruited 2 years post-TBI), delta power was inversely correlated with CRS-R scores. Finally, in the patient who remained in MCS– (recruited 4 years post-TBI), delta power and CRS-R scores were consistently high across all measurement points. Interestingly, a mixed-etiology sample analyzed with a network approach indicated that the prognostic utility of different delta connectivity measures might differ in traumatic and non-traumatic etiologies (45).

In summary, EEG studies of prognosis in chronic DOCs following TBI are extremely limited, both in terms of the number of studies and their sample sizes. In the largest study (14 patients), analyses were further limited by the very small number of patients who recovered from UWS. Importantly, however, these studies highlight that EEG has a potential role in predicting recovery from chronic DOCs, and the metrics of interest (delta power, alpha-derived measures) are likely to be similar to those that correlate with current consciousness level (16, 17). Equally important, these associations were observed in patients who demonstrated clinical improvements several years after the initial TBI, suggesting that ongoing monitoring is important even after the point at which DOCs are often considered permanent (9). Although these sample sizes are very small, it is encouraging to note that similar findings have been reported in mixed-etiology samples that include several patients with TBIs [e.g., (43)]. Nonetheless, the small sample sizes and the diversity of measures used do necessitate caution. It will be necessary to replicate these findings in larger samples, and to compare diverse EEG measures with each other and with standard clinical prognostic tools, before drawing conclusions about the role of EEG for prognostication in chronic DOCs following TBI.

## Discussion

In total, nine studies reported associations between resting-state EEG measures in coma and outcomes during the first year post-TBI (19, 21–23, 25–27). Of these, six reported positive associations with alpha band frequency or alpha-derived measures (19–21, 23–25). Three reported positive associations with theta measures (19, 24, 25). Three reported associations with delta measures [negative: (22); positive: (24, 25)], and two reported positive associations with beta measures (24, 25). One study reported no associations between spectral measures and outcome within days of initial TBI (18). In addition, one study reported positive associations between alpha and beta measures and first-year recovery from UWS (28), and two reported associations between either gamma [negative association; (29)] or alpha and delta measures [positive and negative associations respectively; (30)] with recovery from UWS or MCS, in patients tested months or years after TBI.

Compared to other frequency bands, alpha power is thus most commonly associated with positive prognosis, as might be expected based on its association with an individual's level of consciousness [e.g., (16, 17)]. According to the mesocircuit hypothesis (46), normal alpha activity is generated in the thalamus and reflects the intact functioning of thalamo-cortical loops, which are a prerequisite for consciousness (46–48). When these loops are structurally or functionally disrupted (for example by thalamic damage, or inhibition of the thalamus caused by reductions in background neuronal activity), consciousness is reduced or absent (46). Conversely, the presence of alpha activity indicates intact thalamocortical connections, such that future recovery of consciousness is a possibility (46, 48). In support of this hypothesis it has been noted that damage to the thalamus is common in DOCs, and differentiates UWS from MCS (49). Furthermore, the sedative Zolpidem has the paradoxical effect of increasing consciousness in some patients with MCS by inhibiting the globus pallidus interna, which in turn results in disinhibition of the thalamus and restoration of thalamocortical loop functioning [e.g., (46, 50–52)]. Similarly, stimulation of the thalamus has been linked to behavioral improvements in a patient with MCS (53). The consistency of associations between alpha power and better prognosis noted in this review suggests that, even in the apparent absence of consciousness at the time of recording, alpha activity reflects intact thalamocortical loops, and thus the potential for the brain to support consciousness in the future.

Alpha activity likely also plays a role in shaping the contents of consciousness (48). Once thought to reflect “cortical idling” (54, 55), it is now clear that alpha activity has an active role in perception and cognition, with Jensen and Mazaheri (54) proposing that this role relates to inhibition of task-irrelevant processing. For example, alpha power increases in the primary sensory cortices of task irrelevant sensory modalities, while it decreases in the hemisphere contralateral to targets of attention (54). Likewise, pre-stimulus alpha activity is negatively associated with performance in a motor task, suggesting that it is predictive of lapses in attention before they occur (56). This inhibition may be phasic, with “bouts” of alpha-induced inhibition enhancing perceptual abilities at certain phases of alpha activity (54, 57). Thus, in addition to its significance for intact thalamocortical loops that provide the foundation for consciousness, it is likely that alpha activity also shapes the contents of consciousness *via* its inhibitory role in active perception and cognition. Alpha activity in DOCs may therefore reflect not only intact thalamocortical networks but also the potential for directed attention in a recovering patient.

Although there are clear indications that resting-state EEG measures can have prognostic utility in DOCs following TBI, it is also clear that the literature in this area is incomplete. First, very little is known about the role of EEG in prognosis after the first year; indeed, very little is known about this patient group at all (9). Given the obvious clinical and ethical implications of accurate prognosis for these patients, long-term follow-up studies are a priority. Second, only two studies have evaluated the prognostic value of EEG compared to standard clinical indicators such as GCS scores (20, 26), and only the more recent of these reported an additional benefit (20). Interestingly, Balestreri et al. (58) reported that acute GCS scores and 6-month GOS scores were significantly (though decreasingly) correlated for TBI patients every year between 1992 and 1996, but not significantly correlated between 1997 and 2001. They concluded that GCS scores lost their prognostic value for TBI patients after 1997, and speculated that this might reflect more aggressive pre-hospital treatment, such as sedation and intubation. This further emphasizes the need to ascertain what, if any, additional prognostic value EEG provides in future research. Third, the existing literature has not systematically tested the prognostic value of different EEG measures; many studies have selected only one or two EEG metrics [e.g., (20, 21, 30)]. Some have used more complex metrics, drawn from network approaches, which do not have an intuitive correspondence to the more commonly reported spectral measures (30). Given the more complex analyses required for these approaches, it will be important to assess their advantages over simpler measures. PAV measures (20, 21) are also non-standard, capturing oscillations of alpha activity over hours or days, and thus might be more informative than measures derived from much shorter EEG recordings. A large open-access database of patient resting-state EEG will allow for efficient cross-validation of the prognostic value of multiple and novel EEG features. Fourth, outcome measures have tended to be rather coarse, and might be insensitive to subtle changes in consciousness level. Finally, in chronic DOCs, the literature will benefit from a more systematic consideration of potential confounds, such as time since injury and patient age. Given that patients in these studies will have different diagnoses at baseline (UWS or MCS), it is also important to consider whether final outcome or level of recovery is the most appropriate measure of progress in this group.

## Conclusions

In conclusion, there may be potential for resting-state EEG measures to improve current approaches to prognostication in DOCs following TBI. Based on the existing literature, we suggest that resting-state EEG is particularly promising within the first year post-TBI, and that alpha power and variability are key measures for predicting functional outcome in this time period. However, a more systematic approach, and larger sample sizes, will be required before the clinical value of EEG becomes clear. As a resting-state EEG places no cognitive demands on the patient in question, this approach has potential to identify patients with the residual structural and functional networks required to support consciousness in the future with high sensitivity, thus optimizing subsequent targeting of rehabilitation, therapies, and assessments of both overt and covert cognition. Given the growing evidence for late recovery after TBI, research into prognosis in UWS and MCS, especially after the first year post-TBI, is a priority.

## Author Contributions

RP and AO'D conducted the literature review jointly. RP wrote the manuscript with contributions from AO'D and DC, and oversight from DC. All authors contributed to the article and approved the submitted version.

## Conflict of Interest

The authors declare that the research was conducted in the absence of any commercial or financial relationships that could be construed as a potential conflict of interest.
